# Optimal device selection: Leadless versus conventional transvenous pacemakers

**DOI:** 10.1002/clc.23982

**Published:** 2023-01-25

**Authors:** Naoya Kataoka, Teruhiko Imamura

**Affiliations:** ^1^ Second Department of Internal Medicine University of Toyama Toyama Japan

To the Editor,

Leadless pacemakers have been clinically available in the selected patients. Yu and colleagues compared the impact on patients' quality of life between leadless pacemakers and conventional transvenous pacemakers.^1^ They found that leadless pacemakers were associated with a greater quality of life with fewer activity limitations due to surgical discomfort and less emotional distress. Several concerns have been raised.

The indication of each pacemaker is unclear. A leadless pacemaker might be sufficient in patients with bradycardia‐tachycardia syndrome (sick sinus syndrome type III), given that they are seldom dependent on pacemakers. On the contrary, symptomatic sinus bradycardia (sick sinus syndrome type I) always requires atrial pacing and conventional intravenous pacemakers should be essential. Thus, the device selection might be affected also by the etiologies.

The types of surgery‐related complications are unclear. In their study, the incidence of surgery‐related complications following conventional pacemaker implantation is 21%,^1^ which is relatively higher than the other previous studies. For example, Tjong and colleagues reported 4.7% of device‐related complications following conventional intra‐venous pacemaker implantation and 0.9% following leadless pacemaker implantation.^2^

For the device selection, we should consider also the anatomical feature of each patient. For example, a larger right atrial diameter is associated with longer procedural time during leadless pacemaker implantation.^3^ The leadless pacemaker implantation failed in a patient with an extremely large right atrium.^4^ Thus, we believe that we had better consider several factors other than patients' quality of life for the optimal device selection.

## Data Availability

Not available.
